# *agl*genes, a curated and searchable database of archaeal N-glycosylation pathway components

**DOI:** 10.1093/database/bau046

**Published:** 2014-05-31

**Authors:** Noa Godin, Jerry Eichler

**Affiliations:** Department of Life Sciences, Ben Gurion University of the Negev, P.O. Box 653, Beersheva 84105, Israel

## Abstract

Whereas *N*-glycosylation is a posttranslational modification performed across evolution, the archaeal version of this protein-processing event presents a degree of diversity not seen in either bacteria or eukarya. Accordingly, archaeal *N*-glycosylation relies on a large number of enzymes that are often species-specific or restricted to a select group of species. As such, there is a need for an organized platform upon which amassing information about *a*rchaeal *gl*ycosylation (*agl*) genes can rest. Accordingly, the *agl*genes database provides detailed descriptions of experimentally characterized archaeal *N*-glycosyation pathway components. For each *agl* gene, genomic information, supporting literature and relevant external links are provided at a functional intuitive web-interface designed for data browsing. Routine updates ensure that novel experimental information on genes and proteins contributing to archaeal *N*-glycosylation is incorporated into *agl*genes in a timely manner. As such, *agl*genes represents a specialized resource for sharing validated experimental information online, providing support for workers in the field of archaeal protein glycosylation.

**Database URL:** www.bgu.ac.il/aglgenes

## Introduction

*N*-glycosylation, the covalent attachment of oligosaccharides to selected Asn residues of target proteins, was once thought to be a posttranslational modification unique to eukarya. It has since become clear that bacteria and archaea are also capable of this protein-processing event. However, while bacterial *N*-glycosylation is restricted to certain delta-epsilonproteobacteria strains (1), it appears that this protein modification is a common event in archaea (2). At the same time, archaeal *N*-glycosylation presents variety not seen in the parallel eukaryal or bacterial processes. This diversity is manifested in terms of glycan composition and architecture, the lipid carrier on which the N-linked glycan is assembled and the identity of the sugar that links the glycan to the lipid carrier or the target protein Asn residue (3).

In the past decade, considerable strides have been made in identifying components that catalyze steps of the archaeal *N*-glycosylation process, using genetics, biochemical and mass spectrometry approaches. In particular, detailed *N*-glycosylation pathways based on archaeal glycosylation (Agl) proteins have been delineated in the halophile *Haloferax volcanii* and in the methanogens *Methanococcus maripaludis* and *Methanococcus voltae* (3–6). Insight into the process of *N*-glycosylation in the thermoacidophile *Sulfolobus acidocaldrius* has also been provided (7). For the most part, these pathways rely on species-specific pathway components or components restricted to a group of species. Examination of the limited number of archaeal N-linked glycans for which structural information has been reported points to the existence of numerous different *N*-glycosylation pathways (3). Given that the recent analysis of 168 available archaeal genomes has identified genes encoding AglB, the oligosaccharyltransferase central to *N*-glycosylation, in 166 cases (2), it would appear that the list of known archaeal *N*-glycosylation pathway components is only the tip of an iceberg.

With the aim of collecting all of the experimentally obtained information on archaeal *N*-glycosylation pathway components into a single location that will allow comparative analysis, we have assembled the *agl*genes database and created a Web site that allows users access to the information contained in the database. The database allows users to search according to species, protein class or gene name to obtain all currently available genomic, biochemical, structural and functional information on archaeal *N*-glycosylation pathway components. The routinely updated *agl*genes database can be found at www.bgu.ac.il/aglgenes.

## Methods and usage

### Aims of the database

The primary aims of the *agl*genes database are to list all available experimental information on *agl* (*a*rchaeal *gl*ycosylation) genes and proteins that are involved in archaeal *N*-glycosylation pathways and to provide this information in a user-friendly web interface.

### Methods

**Figure 1.**
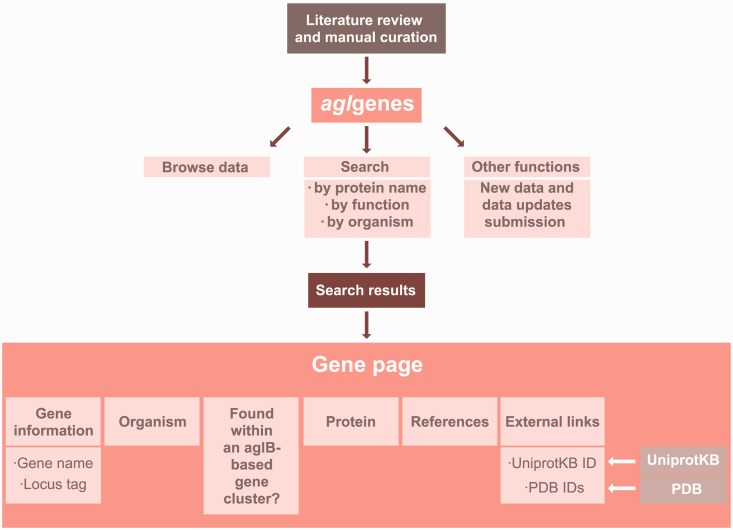
A detailed illustration of the *agl*genes database architecture.

All available experimental data pertaining to the components of archaeal *N*-glycosylation pathways or individual enzymes shown to be involved in archaeal *N*-glycosylation were collected from the literature and manually curated. The data are stored and maintained in a relational database using the MySQL database management system (http://www.mysql.com/). A specific naming convention was established to uniquely identify each *agl* gene entry. Each gene ID starts with the letters ‘agEL’ followed by two digits (e.g. agEL05). The Web site interface uses embedded MySQL queries, PHP5 code to execute the queries and HTML5–CSS3 code to format the query results. New information will be added to the Web site on a monthly basis or whenever such information becomes available. A complete layout of the architecture of the *agl*genes database is presented in Figure 1.

### Usage

**Figure 2.**
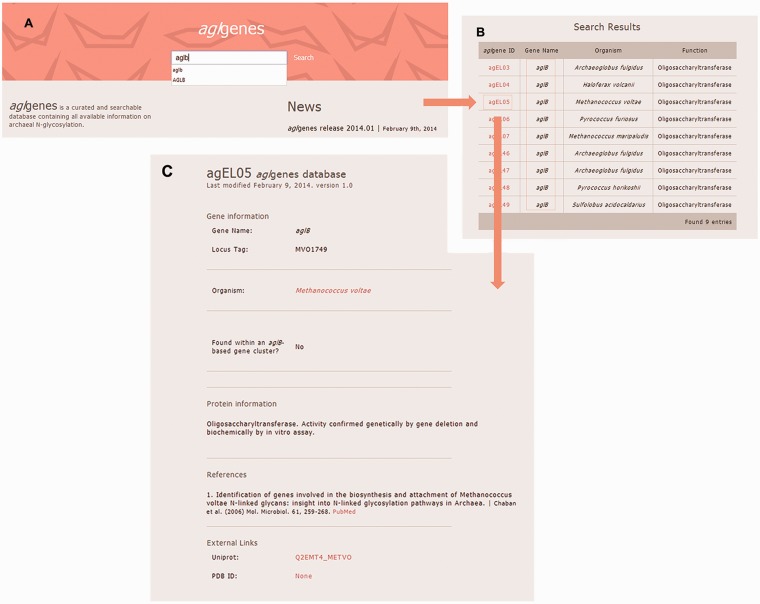
Screenshots of the results of a sample *agl*genes query. (**A**) The user inputs the string ‘aglb’ in the search field, for example. (**B**) The results for the query are presented in tabular format. (**C**) After selecting the entry of interest from the list of organisms containing the gene selected in this example, detailed information for the selected *agl*genes entry is retrieved in a new web page.

*agl*genes provides the user with an intuitive web interface that does not require any particular training. The latest version of any web browser is recommended for the usage of the *agl*genes database. The database includes a single search field that allows the user to search through all published experimental data on genes and proteins involved in archaeal *N*-glycosylation, according to gene name or protein function. Alternatively, users can retrieve all known *N*-glycosylation pathway components from a given archaeal species. The query results are displayed in a tabular format with the gene’s *agl*genes ID, name, function and source organism. Clicking the *agl*genes ID link opens a new web page containing all information for the selected *agl* gene. The results of a sample query are featured in Figure 2.

Each retrieved *agl*genes gene entry includes gene information, such as gene name and locus tag, the organism(s) containing the gene and available functional information on the protein product. In addition, each entry indicates whether the gene of interest is found as part of an *aglB*-based *N*-glycosylation gene cluster. AglB, the archaeal oligosaccharyltransferase responsible for transfer of a glycan from the lipid carrier on which it is assembled to target protein Asn residues (4, 8, 9), is a central component of the archaeal *N*-glycosylation process. Other details, such as genomic context and relevant references, are also provided for each *agl*genes gene entry. Links to external sources, such as Uniprot, NCBI and PDB, are also provided for each entry according to availability. Table 1 lists all fields in a given *agl*genes entry and their descriptions.

**Table 1.** Description of fields used to annotate an *agl*gene entry

Field name	Description
ID	Unique *agl*genes identifier
Gene information	
Gene name	Approved gene name
Locus tag	Approved gene locus identifier
Organism	*agl*genes entry source organism
Found within an *aglB*-based gene cluster?	Indicates whether the *agl* gene is a part of a cluster containing an *aglB* gene in the specific organism
Protein information	Protein function and summary of existing experimental information
Genomic context	Genomic context of the *agl* gene
References	Papers reporting the *agl* gene and other relevant *agl*genes information
External links	
UniprotKB ID	External link to Uniprot KB
Protein DataBank ID	External link to Protein DataBank

Finally, an online data submission page can also be found at the *agl*genes database Web site to allow users to submit updates on existing entries or new information on archaeal *N*-glycosylation pathway components.

## Conclusions

Genome-based studies suggest that *N*-glycosylation is a common posttranslational modification in Archaea (2). At the same time, because sequence-based analysis can only provide limited insight into the precise function of sugar-processing enzymes, deciphering archaeal *N*-glycosylation pathways has been limited to a restricted number of species for which appropriate molecular tools are available (3, 6, 7). However, as an increasing number of different archaeal species are being adopted as model systems, the techniques and tools required for detailed delineation of *N*-glycosylation pathway components are starting to appear. Such efforts have revealed the largely species-specific nature of *N*-glycosylation pathway components. Indeed, given the enormous variety seen in terms of the content and structure of even the few characterized *N*-linked glycans decorating archaeal glycoproteins the recruitment of species-specific pathways is not surprising. On the other hand, as more data accumulate, it seems that certain sugar-processing enzymes are involved in *N*-glycosylation pathways in more than one organism. As a repository of all available experimentally confirmed information on archaeal *N*-glycosylation, the *agl*genes database allows researchers interested in archaea and/or in *N*-glycosylation to learn more about this posttranslational modification that, in some cases, is associated with the ability of these organisms to survive the extreme environmental conditions they encounter (10).

## Funding

Israel Science Foundation (grant 8/11) and the US Army Research Office (W911NF-11-1-520). Funding for open access charge: Israel Science Foundation.

*Conflict of interest*. None declared.
